# Response to anaplastic lymphoma kinase inhibitor in gastric cancer harboring *DCTN1–ALK* fusion: a case report and review

**DOI:** 10.3389/fimmu.2025.1686666

**Published:** 2025-10-30

**Authors:** Huadi Wang, Liangkun You, Hong Pan, Xiaotong Qiu, Jin Sheng

**Affiliations:** Department of Medical Oncology, Zhejiang University School of Medicine Sir Run Run Shaw Hospital, Hangzhou, China

**Keywords:** anaplastic lymphoma kinase (ALK), circulating tumor DNA (ctDNA), next generation sequencing (NGS), case report, review

## Abstract

Anaplastic lymphoma kinase (*ALK*) rearrangements are exceedingly rare in gastric cancer, and uncommon fusion types add to the difficulties of proper, precise treatment strategies. Although detected in non-small cell lung cancer (NSCLC), inflammatory myofibroblastic tumors (IMTs), and Spitz tumors, the *DCTN1–ALK* fusion has not previously been reported in gastric cancer. This report describes the first case of gastric adenocarcinoma harboring a *DCTN1–ALK* fusion that was successfully treated with the ALK-targeted agent alectinib after first- and second-line chemotherapy-based regimens had failed. Progression-free survival on alectinib was 11.5 months until KRAS amplification emerged on serial circulating tumor DNA analysis, leading to rapid systemic relapse. The other documented cases with *DCTN1–ALK* fusion treated with the first or second generation of ALK inhibitors indicated this rare fusion as an actionable driver gene mutation. This successful personalized anti-tumor strategy highlights the clinical utility of comprehensive genomic profiling and liquid biopsy in detecting and monitoring actionable *ALK* fusions in solid tumors.

## Introduction

Gastric cancer still poses a significant challenge in oncology due to its heterogeneity and unsatisfactory response to traditional chemotherapy. Recent advancements in cancer research have identified gastric cancer as subgroups based on epidemiologic (1), genomic (2), or molecular (3) classifications that may benefit from potential therapeutic targets. Among the various subtypes of gastrointestinal (GI) cancers, although very rare, anaplastic lymphoma kinase (*ALK*) rearrangements represent a particularly intriguing group, suggesting a potential for targeted therapy with ALK inhibitors (ALKi), offering a ray of hope for personalized treatment approaches.

The *ALK* gene encodes a receptor tyrosine kinase that is involved in cell growth, proliferation, and survival. Mutations in the *ALK* gene can lead to the aberrant activation of the ALK protein, resulting in uncontrolled cell growth and tumor formation. *ALK* rearrangements are extremely rare in GI cancers, with a frequency of <1% in colorectal cancer (4), 0.2% in pancreatic cancer (5), and 0.9% to 2.3% in gastric cancer (1, 6), but may offer personalized treatment strategies in selected patients. A Korean gastric cancer cohort (n = 455) consisted of 38 ALK Immunohistochemistry (IHC)-positive (34 of 1+, and 4 of 2+/3+) patients who were younger and more likely to have signet ring cells (1), associated with worse disease-free survival (DFS) and overall survival (OS) (1). Fortunately, ALKi, such as alectinib and lorlatinib (7), have demonstrated efficacy in inhibiting tumor growth and improving patient outcomes in *ALK*-mutant gastric cancer, offering a more effective and less toxic alternative to traditional chemotherapy.

The appearance of the *DCTN1–ALK* fusion gene in cancer is a rare genetic rearrangement event primarily associated with the occurrence and progression of tumors. Dynactin subunit 1 (DCTN1) is a protein related to the cytoskeleton, involved in the transport of materials within the cell. When *DCTN1* undergoes a gene fusion with *ALK*, *DCTN1* is suspected to promote the dimerization of *ALK* and lead to the abnormal activation of the ALK kinase by transphosphorylation, thereby promoting the proliferation and survival of tumor cells (8). To date, published case reports of *DCTN1–ALK* fusion solid tumors included non-small cell lung cancer (NSCLC) (9–11), inflammatory myofibroblastic tumors (IMTs) (12, 13), Spitz tumors (14), and some rare types of neoplasms.

Given the rarity of both *ALK* mutation and the fusion type, here, we report a case of a gastric cancer patient with a driver mutation of *DCTN1–ALK* fusion. Although the first and second lines of standard chemotherapy ended in progression of disease, subsequent targeted therapy improved the general health of the patient and prolonged the survival.

## Case presentation

**Figure 1:**
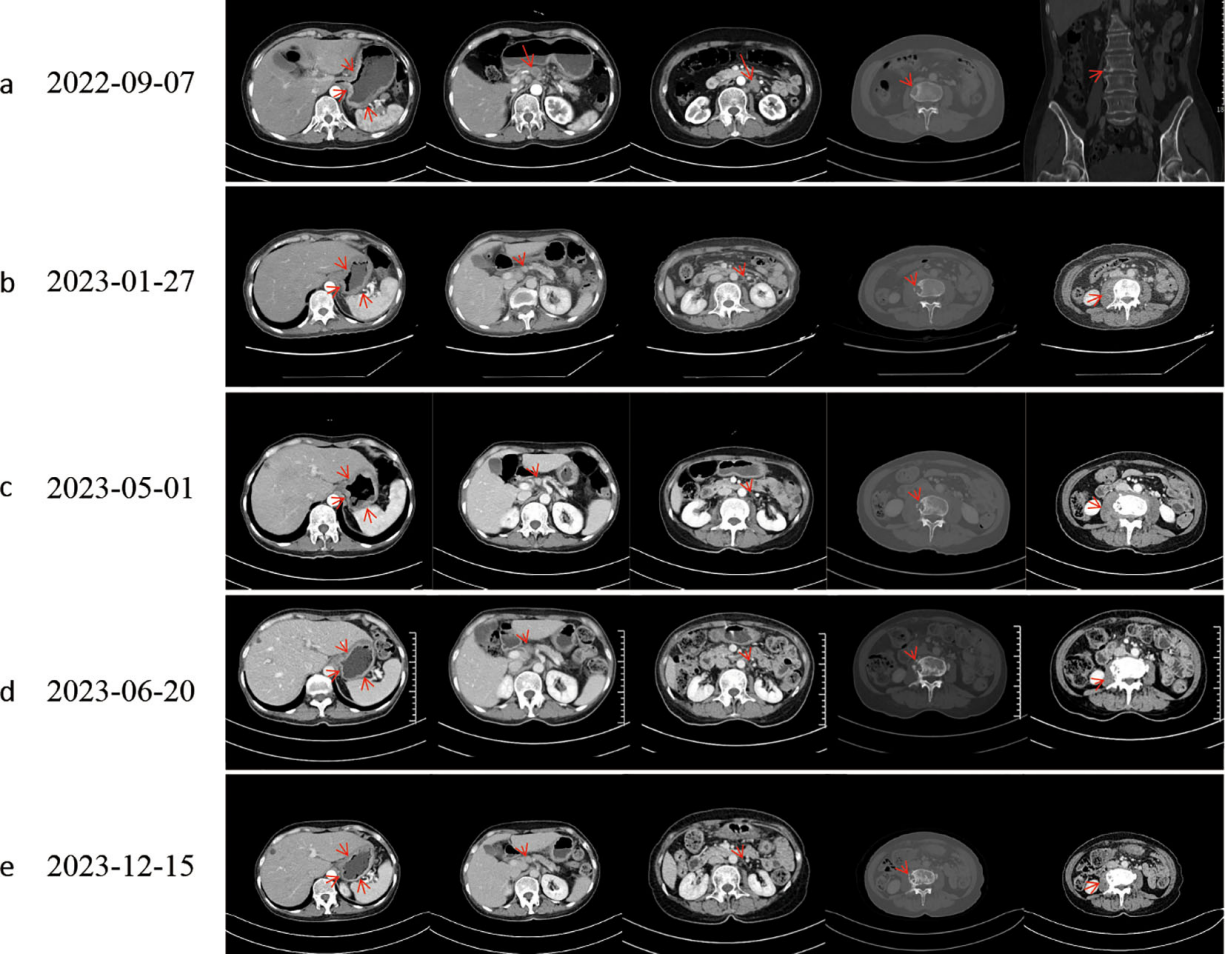
Computed tomography evaluation. Following effective therapy, the patient exhibited decreased gastric wall thickness, reduced osteolytic destruction of bone metastases, increased osteoblastic changes, and diminished perilesional soft-tissue components around osseous metastases. **(a)** Baseline. **(b)** Evaluation after progression of first-line chemotherapy. **(c)** Evaluation after progression of second-line chemotherapy; L3 vertebral metastasis showed progressive osteolytic destruction with an expanding perilesional soft-tissue mass. **(d, e)** The best response to targeted therapy was a partial response (PR).

**Figure 2:**
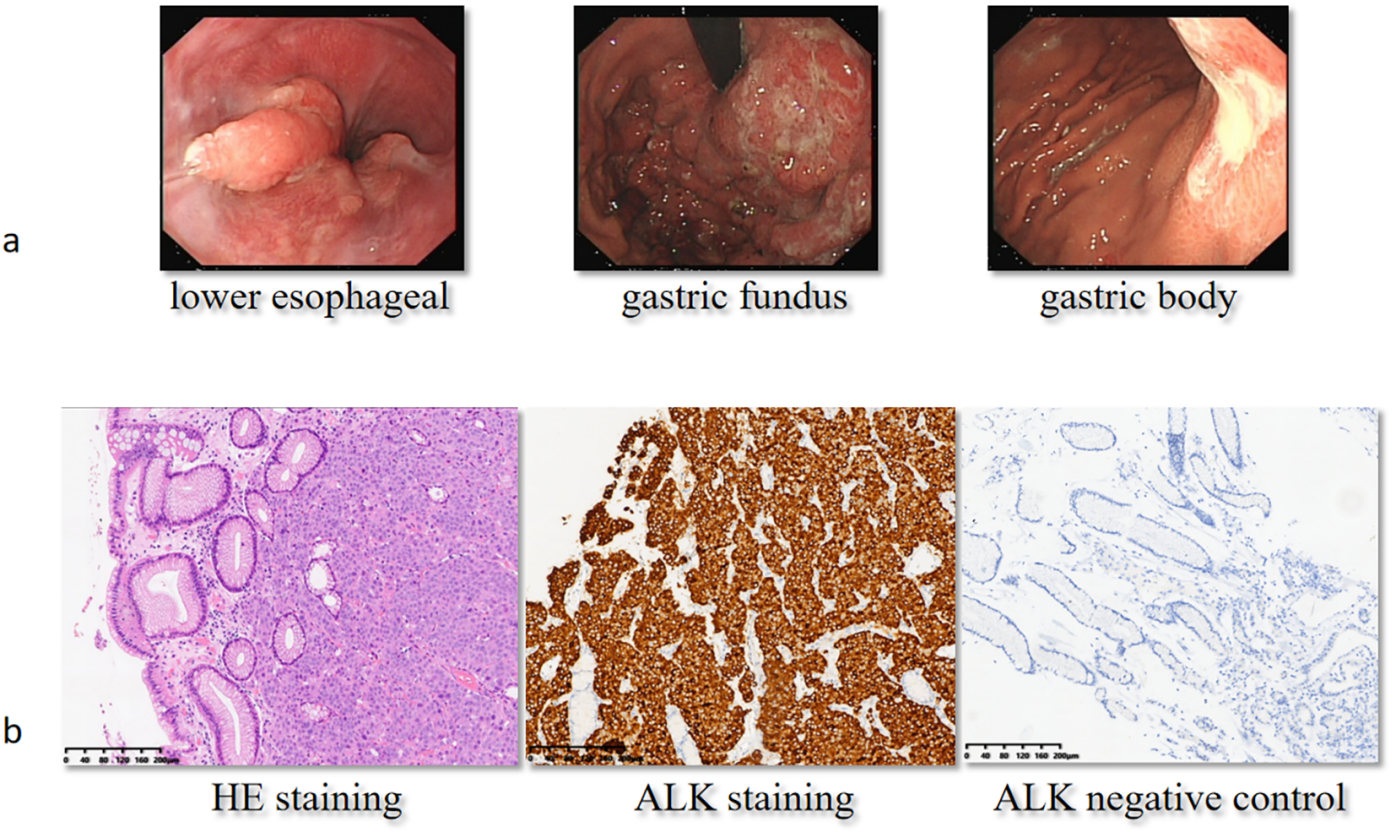
Gastroduodenoscopy and immunohistochemical analysis at baseline. On September 8, 2022, the patient received a gastroduodenoscopy **(a)**, and the pathologist reported poorly differentiated carcinoma with positive ALK staining **(b)**. H&E, hematoxylin and eosin; ALK, anaplastic lymphoma kinase.

The patient, a 58-year-old woman, was admitted to the local hospital due to symptoms of cough, decline in appetite, and mild weight loss in August 2022. Laboratory tests revealed a significant increase in carcinoembryonic antigen (CEA) to 323.26 ng/mL and CA724 to 266.83 U/mL. The patient was then transferred to the Department of General Practice. Physical examinations did not show positive findings. A chest computed tomography (CT) scan showed enlarged mediastinal lymph nodes. An enhanced abdominal CT scan revealed thickening with enhancement of the lateral wall of the gastric cardia, fundus, and lesser curvature of the gastric body, indicating a T4a malignant tumor (**Figure 1a**). In addition, L3 vertebral metastasis was suspected. Multiple lymph node enlargements were observed in the perigastric, hilar, mesenteric, and retroperitoneal regions, consistent with metastasis (cT4aN3M1, stage IV). On September 8, 2022, the patient received a gastroduodenoscopy, and biopsies of the gastric angle, fundus, and lower esophagus were pathologically diagnosed as poorly differentiated carcinoma (**Figure 2**). Immunohistochemical analysis showed positive staining for cytokeratin (CK7), cytokeratin pan-cocktail (AE1/AE3), chromogranin A (focal positive), Ki-67 (40%), and ALK (D5F3) (**Figure 2**), and negative staining for S-100, thyroid transcription factor 1 (TTF1), hepatocyte, estrogen receptor, synaptophysin (Syn), CD56, CD3, CD20, GATA-3, NapsinA, SOX10, and human epidermal growth factor receptor 2 (HER2) (**Supplementary Figure 1**), as well as intact expression of mismatch repair proteins.

### Standard chemotherapy phase

From September 20, 2022, to December 14, 2022, the patient received chemotherapy combined with immunotherapy for five cycles, consisting of capecitabine and oxaliplatin (XELOX) plus sintilimab. Meanwhile, a whole-body bone CT scan on November 22, 2022, showed multiple abnormal increases in bone metabolism at the seventh cervical vertebra, 10th and 11th thoracic vertebrae, third lumbar vertebra, right third anterior rib, sacrum, bilateral iliac bones, right ischium, and upper segment of the left femur, primarily indicating tumor bone metastasis. The patient received bisphosphonate therapy to retard osteolysis.

**Figure 3:**
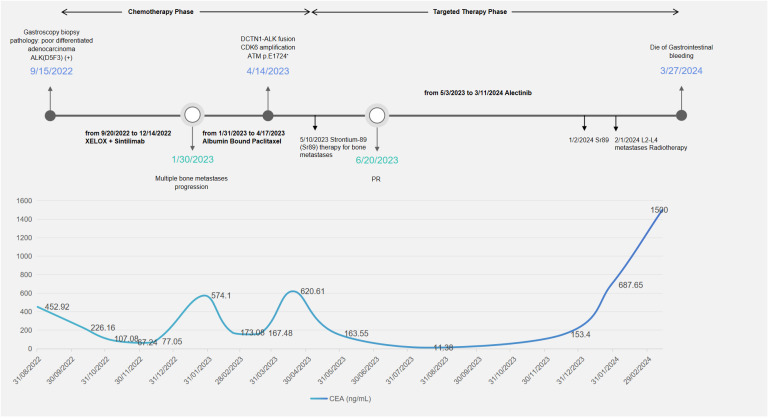
Timeline of treatment and CEA changes. During the chemotherapy phase, the patient received two lines of treatment: XELOX plus sintilimab and albumin-bound paclitaxel. The patient received strontium-89 therapy and localized radiotherapy for bone metastases. XELOX, capecitabine and oxaliplatin; PR, partial response; CEA, carcinoembryonic antigen.

On January 27, 2023, due to elevated tumor markers (**Figure 3**) and increased soft tissue at the L3 metastatic lesion (**Figure 1b**), the disease was considered to be progressing. From January 31, 2023, to April 17, 2023, the patient received single-agent chemotherapy with albumin-bound paclitaxel as second-line treatment.

### Targeted therapy phase

During second-line chemotherapy, the patient’s CEA level decline remained suboptimal (**Figure 3**), indicating a limited therapeutic response (**Figure 1c**); consequently, the patient opted for next-generation sequencing (NGS)-based genomic profiling. On April 14, 2023, NGS gene testing was completed, revealing a *DCTN1–ALK* gene fusion, *ATM* p.E1724*, and amplification of the *CDK6* gene by hybrid-capture NGS performed on DNA extracted from the gastric-biopsy formalin-fixed paraffin-embedded (FFPE) tissue on September 8, 2022. On May 3, 2023, the patient received targeted therapy with alectinib. On May 10, 2023, the patient underwent strontium-89 (Sr-89) therapy for metastatic bone tumor control and severe pain relief. During follow-up, the patient demonstrated excellent disease control: CT revealed gastric wall thinning, and the previously lytic L3 metastasis exhibited osteogenic changes (**Figures 1d, e**).

### Post-resistance phase

In January 2024, surveillance revealed a marked surge in CEA, suggesting incipient, gradual resistance to targeted therapy. Subsequent MRI of the L3 vertebra revealed epidural spinal cord compression from osseous metastasis, prompting palliative radiotherapy to the L2–L4 metastatic lesion in February 2024. The whole blood circulating tumor DNA (ctDNA) gene test revealed the disappearance of the original *DCTN1–ALK* fusion and the concomitant emergence of a *de novo KRAS* amplification mutation. The patient subsequently experienced fulminant systemic progression and ultimately died of uncontrollable gastrointestinal hemorrhage.

## Discussion

*ALK* rearrangements occur at low prevalence in a spectrum of non-pulmonary solid tumors, yet they have emerged as actionable, pan-cancer driver events among solid neoplasms. Activating *ALK* alterations—comprising gain-of-function point mutations, high-level gene amplifications, and oncogenic fusions/rearrangements—have been documented in a spectrum of malignancies that includes NSCLC, anaplastic large-cell lymphoma (ALCL; accounting for approximately 0.5% of adult lymphomas and roughly 10% of pediatric non-Hodgkin lymphomas), IMT, neuroblastoma, cutaneous spitzoid neoplasms, and inflammatory breast carcinoma. Population-level genomic profiling identifies *ALK* alterations in ~3.3% of all cancers, with fusions/rearrangements representing a minority subset (~0.5%–0.8%) (15). Within NSCLC, the prevalence of *ALK* rearrangements exceeds 3%, whereas non-NSCLC malignancies exhibit a markedly lower incidence (~0.2%). Beyond NSCLC, IMT (~50%) and ALCL (50%–80%) are the entities most frequently driven by *ALK* fusions. While the overwhelming majority of *ALK*-positive NSCLCs harbor an *EML4–ALK* fusion (83.5%), this chimeric transcript is encountered in only ~31% of *ALK*-rearranged non-NSCLC tumors, which instead display marked heterogeneity in 5′ fusion partners (16). In gastric cancer, *ALK* fusions reported involve *RAB10* (17) or *HMBOX* (7); here, we report the first case of *DCTN1–ALK* fusion.

The appearance of the *DCTN1–ALK* fusion gene in cancer is a rare genetic rearrangement event, primarily associated with the occurrence and progression of tumors. DCTN1 is a protein related to the cytoskeleton, involved in the transport of materials within the cell (18). When *DCTN1* undergoes a gene fusion with *ALK*, it may lead to the abnormal activation of the ALK kinase, thereby promoting the proliferation and survival of tumor cells. To date, the *DCTN1–ALK* fusion gene has been reported in three cases of NSCLC, one case of IMT (12), six cases of Spitz tumors (14), one case of epithelioid fibrous histiocytoma (19), and one case of pancreatic tumors (20).

With the advancement of NGS technology, rare *ALK* fusion subtypes, such as *DCTN1–ALK*, are being increasingly identified, which has facilitated the development of more precise, targeted treatment strategies for patients with these rare fusions. All published cases have confirmed a clinical response to targeted therapy in tumors harboring the *DCTN1–ALK* fusion protein. Among the three lung cases, the lung cancer patient (9) with a single *DCTN1–ALK* mutation received crizotinib orally at a dose of 250 mg twice daily, which resulted in a significant symptomatic improvement and radiographic response after 3 months of therapy. The female lung adenocarcinoma patient (10) (concurrent *EGFR* mutations and *DCTN1–ALK* fusion) with brain metastases developed resistance to chemotherapy or targeted therapy. CtDNA was dynamically monitored and confirmed the coexistence of a primary *EGFR T790M* mutation/*EGFR* exon 19 deletion and *DCTN1–ALK* translocation. She responded to osimertinib and alectinib treatment, and after acquiring osimertinib resistance, the patient still responded to alectinib and achieved a partial response (PR) for lung and brain lesions. The lung adenocarcinoma patient (11) harboring dual *DCTN1–ALK* and *ALK-CLIP4* rearrangements showed PR to crizotinib with a 12-month progression-free survival (PFS) and received alectinib as second-line treatment. In the IMT case (12), the patient, who had confirmed a diagnosis of a *DCTN1–ALK* fusion through genetic profiling, was enrolled in a phase I clinical trial (ClinicalTrials.gov Identifier: NCT01548144) of crizotinib in combination with pazopanib. The patient was treated with crizotinib 250 mg orally on alternating days and pazopanib 200 mg orally daily and had confirmed PR for over 6 months. In our gastric adenocarcinoma case, the chemotherapy phase lasted for approximately 8 months. During chemotherapy, the primary gastric lesion demonstrated a limited response to chemotherapy +/− immunotherapy. However, the bone metastases exhibited primary resistance, resulting in severe pain and impairment of activities of daily living. On the contrary, alectinib targeted therapy reached PR, and the PFS reached 11.5 months.

In the other two cases of *ALK* fusion gastric cancer, one with *RAB10–ALK* mutation refused off-label use of agents such as crizotinib and ceritinib, and the other patient with *ALK–HMBOX* fusion received alectinib as first-line targeted therapy and achieved complete response (CR) in thoracic and cervical metastatic lymph nodes and PR in brain metastases, which reached a 6-month PFS. The second-line targeted therapy of lorlatinib also showed a 6-month PFS, with the best overall response of stable disease (SD). Likewise, other rare *ALK*-altered digestive system neoplasms confront therapeutic decision challenges. In a case series report of 13 GI cancer patients (7), regardless of the prior standard systemic treatment, ALKi chosen as the first-line target agent were alectinib for seven patients, crizotinib for five patients, and entrectinib for one patient. Further lines of ALKi included alectinib, lorlatinib, and ceritinib. Ten of 12 evaluable patients (83%) achieved a PR or SD response, and the median PFS was 5.0 months; the median OS was 9.3 months. Overall, patients with non-pulmonary malignancies harboring *ALK* alterations exhibited shorter PFS on targeted therapy than those with *ALK*-positive lung cancer. The breakpoint identified in our case with baseline tumor specimen (*DCTN1-E19:ALK-E20*) mirrors that described in NSCLC, implying that the shorter progression-free survival observed in GI malignancies does not merely depend on structural differences in the fusion itself but may be comprehensively affected by patient-related tumor microenvironment or co-occurring genomic events, such as *CDK6* amplification and *ATM* mutation.

In summary, *DCTN1–ALK* fusion behaves biologically like other gastric *ALK* fusions [same kinase domain, equal *in vitro* Tyrosine kinase Inhibitor (TKI) sensitivity], but three features appear unique to the *DCTN1* partner. First, *DCTN1–ALK* retains the same ALK kinase domain and *in vitro* drug sensitivity as other gastric *ALK* fusions, indicating equal intrinsic signaling potency. Second, it reproducibly associates with poorly differentiated histology and bone metastases, suggesting a phenotypic footprint linked to the *DCTN1* cytoskeletal role. Third, our case and the published lung tumors frequently harbor co-alterations such as *ATM* loss or *CDK6* amplification that shorten TKI durability. Taken together, the fusion is not inherently “stronger”, but its biological distinctiveness lies in histology, tropism, and genomic context, supporting comprehensive gene profiling in specific situations, especially for those with refractory gastric cancer. Therefore, based on an actionable fusion frequency ≥0.5%, a clinically relevant response rate to ALK TKIs, and marginal additional cost when incorporated into existing comprehensive NGS workflows, we advocate routine *ALK* fusion assessment in all cases of metastatic or refractory gastric cancer.

Dynamic genomic profiling to monitor resistance mechanisms can guide precise regimen adjustments. Given that rare variants lack evidence-based therapeutic guidance and the challenges of repeat tissue biopsies, liquid biopsy options, such as peripheral blood ctDNA testing, offer a practical alternative. Several cases have reported the use of dynamic ctDNA sequencing to identify resistance mechanisms after first-line targeted therapy, thereby informing subsequent targeted treatment selection. In the case of carcinoma of unknown primary with an *EML4–ALK* fusion (21), ctDNA detection revealed two resistance mutations (L1196M and G1269A) to crizotinib. Therefore, the targeted therapy was switched to brigatinib 180 mg once a day p.o., and CT examination confirmed PR. The gastric cancer case with *ALK–HMBOX* fusion failed to reveal the resistance mechanism by a second FoundationOne CDx test on the new cervical lymph node tissue specimen; however, the p.Val1180Leu ALKi resistance mutation was identified in ctDNA analyzed on a Illumina NextSeq 550 NGS instrument. Then, the patient started lorlatinib 50 mg twice a day as second-line treatment. After progression, an *STK11* intronic loss-of-function mutation (c.734 + 1G.T) was detected as a resistance mechanism through liquid biopsy using pleural effusion and plasma. In our case, the *DCTN1–ALK* fusion was first detected in the primary gastric tissue specimen; cfDNA analysis was employed later for longitudinal surveillance and revealed the emergence of *KRAS* amplification at progression. It is supposed that the *KRAS* amplification mutation replaced *DCTN1–ALK* fusion to emerge as the driver gene, precipitating rapid and widespread tumor progression. The patient ultimately lost all further anti-tumor therapeutic opportunities. Although the index detection of *DCTN1–ALK* was tissue-based, subsequent resistance profiling relied on cfDNA; therefore, we cannot determine whether the *KRAS*-amplified sub-clone originated from the primary tumor, bone metastases, or both.

To date, a limited number of basket trials have been initiated to investigate better targeted therapy strategies and resistance mechanisms in patients with *ALK*-altered non-pulmonary malignancies. There are several ongoing basket trials listed on the ClinicalTrials.gov website, with identifiers NCT04644315, NCT03868423, NCT01284192, and NCT04439266 (7). With continuing advances and innovations in gene sequencing technologies, we anticipate an expansion of regional and global basket trials that will establish a robust evidence base for precision-targeted therapies against rare *ALK* alterations.

## Conclusion

Despite the progress made in understanding and targeting *ALK* fusions in non-lung cancers, challenges remain in optimizing treatment strategies and overcoming resistance to ALK inhibitors. GI cancer patients harboring *ALK* mutations are at risk of being excluded from personalized treatment, which may dramatically improve survival due to the conventional test and treatment routine. To our knowledge, this is the first case report of gastric cancer harboring *DCTN1–ALK* fusion with clinical response to targeted therapy. Liquid biopsy plays a pivotal role in the dynamic genomic surveillance of patients in real-world clinical practice. Further research is needed to identify novel therapeutic combinations that can improve patient outcomes and elucidate the mechanisms of resistance to *ALK*-driven solid tumors.

## Data Availability

The original contributions presented in the study are included in the article/**Supplementary Material**. Further inquiries can be directed to the corresponding author.

## References

[1] Chon HJ Kim HR Shin E Kim C Heo SJ Lee C . The clinicopathologic features and prognostic impact of ALK positivity in patients with resected gastric cancer. Ann Surg Oncol. (2015) 22:3938–45. doi: 10.1245/s10434-015-4376-8, PMID: 25707491

[2] Deng N Goh LK Wang H Das K Tao J Tan IB . A comprehensive survey of genomic alterations in gastric cancer reveals systematic patterns of molecular exclusivity and co-occurrence among distinct therapeutic targets. Gut. (2012) 61:673–84. doi: 10.1136/gutjnl-2011-301839, PMID: 22315472 PMC3322587

[3] Shah MA Khanin R Tang L Janjigian YY Klimstra DS Gerdes H . Molecular classification of gastric cancer: A new paradigm. Clin Cancer Res. (2011) 17:2693–701. doi: 10.1158/1078-0432.CCR-10-2203, PMID: 21430069 PMC3100216

[4] Aisner DL Nguyen TT Paskulin DD Le AT Haney J Schulte N . *ROS1* and *ALK* fusions in colorectal cancer, with evidence of intratumoral heterogeneity for molecular drivers. Mol Cancer Res. (2014) 12:111–8. doi: 10.1158/1541-7786.MCR-13-0479-T, PMID: 24296758 PMC4140177

[5] Singhi AD Ali SM Lacy J Hendifar A Nguyen K Koo J . Identification of targetable *ALK* rearrangements in pancreatic ductal adenocarcinoma. J Natl Compr Canc Netw. (2017) 15:555–62. doi: 10.6004/jnccn.2017.0058, PMID: 28476735

[6] Alese OB El-Rayes BF Sica G Zhang G Alexis D La Rosa FG . Anaplastic lymphoma kinase (ALK) gene alteration in signet ring cell carcinoma of the gastrointestinal tract. Ther Adv Med Oncol. (2015) 7:56–62. doi: 10.1177/1758834014567117, PMID: 25755678 PMC4346214

[7] Ambrosini M Del Re M Manca P Hendifar A Drilon A Harada G . ALK inhibitors in patients with ALK fusion-positive GI cancers: an international data set and a molecular case series. JCO Precis Oncol. (2022) 6:e2200015. doi: 10.1200/PO.22.00015, PMID: 35476549 PMC9200393

[8] Chiarle R Voena C Ambrogio C Piva R Inghirami G . The anaplastic lymphoma kinase in the pathogenesis of cancer. Nat Rev Cancer. (2008) 8:11–23. doi: 10.1038/nrc2291, PMID: 18097461

[9] Vendrell JA Taviaux S Béganton B Godreuil S Audran P Grand D . Detection of known and novel ALK fusion transcripts in lung cancer patients using next-generation sequencing approaches. Sci Rep. (2017) 7:12510. doi: 10.1038/s41598-017-12679-8, PMID: 28970558 PMC5624911

[10] Yin Q Guo T Zhou Y Sun L Meng M Ma L . Effectiveness of alectinib and osimertinib in a brain metastasized lung adenocarcinoma patient with concurrent * EGFR * mutations and * DCTN1-ALK * fusion. Thorac Cancer. (2022) 13:637–42. doi: 10.1111/1759-7714.14291, PMID: 34964276 PMC8841708

[11] Gao F Gao F Wu H Lu J Xu Y Zhao Y . Response to ALK-TKIs in a lung adenocarcinoma patient harboring dual * DCTN1-ALK * and * ALK-CLIP4 * rearrangements. Thorac Cancer. (2022) 13:1088–90. doi: 10.1111/1759-7714.14345, PMID: 35212154 PMC8977168

[12] Subbiah V McMahon C Patel S Zinner R Silva EG Elvin JA . STUMP un”stumped”: anti-tumor response to anaplastic lymphoma kinase (ALK) inhibitor based targeted therapy in uterine inflammatory myofibroblastic tumor with myxoid features harboring DCTN1-ALK fusion. J Hematol Oncol. (2015) 8:66. doi: 10.1186/s13045-015-0160-2, PMID: 26062823 PMC4467062

[13] Denis D Elayoubi K Weil A Berthelet F Bojanowski M . Inflammatory myofibroblastic tumors of the central nervous system that express anaplastic lymphoma kinase have a high recurrence rate. Surg Neurol Int. (2013) 4:70. doi: 10.4103/2152-7806.112614, PMID: 23776756 PMC3683168

[14] Wiesner T He J Yelensky R Esteve-Puig R Botton T Yeh I . Kinase fusions are frequent in Spitz tumours and spitzoid melanomas. Nat Commun. (2014) 5:3116. doi: 10.1038/ncomms4116, PMID: 24445538 PMC4084638

[15] The AACR Project GENIE Consortium, The AACR Project GENIE Consortium André F Arnedos M Baras AS Baselga J Bedard PL . AACR project GENIE: powering precision medicine through an international consortium. Cancer Discov. (2017) 7:818–31. doi: 10.1158/2159-8290.CD-17-0151, PMID: 28572459 PMC5611790

[16] Ross JS Ali SM Fasan O Block J Pal S Elvin JA . *ALK* fusions in a wide variety of tumor types respond to anti-ALK targeted therapy. Oncol. (2017) 22:1444–50. doi: 10.1634/theoncologist.2016-0488, PMID: 29079636 PMC5728036

[17] Wen Z Xiong D Zhang S Liu J Li B Li R . Case report: RAB10-ALK: A novel ALK fusion in a patient with gastric cancer. Front Oncol. (2021) 11:645370. doi: 10.3389/fonc.2021.645370, PMID: 33692962 PMC7938725

[18] Lee YD Kim B Jung S Kim H Kim MK Kwon J-O . The dynactin subunit DCTN1 controls osteoclastogenesis via the Cdc42/PAK2 pathway. Exp Mol Med. (2020) 52:514–28. doi: 10.1038/s12276-020-0406-0, PMID: 32210358 PMC7156411

[19] Dickson BC Swanson D Charames GS Fletcher CD Hornick JL . Epithelioid fibrous histiocytoma: molecular characterization of ALK fusion partners in 23 cases. Modern Pathol. (2018) 31:753–62. doi: 10.1038/modpathol.2017.191, PMID: 29327718

[20] Shimada Y Kohno T Ueno H Ino Y Hayashi H Nakaoku T . An oncogenic *ALK* fusion and an *RRAS* mutation in *KRAS* mutation-negative pancreatic ductal adenocarcinoma. Oncol. (2017) 22:158–64. doi: 10.1634/theoncologist.2016-0194, PMID: 28167572 PMC5330701

[21] Zhao P Peng L Wu W Zheng Y Jiang W Zhang H . Carcinoma of unknown primary with *EML4-ALK* fusion response to *ALK* inhibitors. Oncol. (2019) 24:449–54. doi: 10.1634/theoncologist.2018-0439, PMID: 30679319 PMC6459257

