# Supplementary material for: Response to anaplastic lymphoma kinase inhibitor in gastric cancer harboring DCTN1–ALK fusion: a case report and review
Source: Front Immunol. 2025 Oct 30;16:1686666. doi: 10.3389/fimmu.2025.1686666 (PMC12611845; doi:10.3389/fimmu.2025.1686666)
Supplement: Supplementary file 1 [file DataSheet1.pdf]

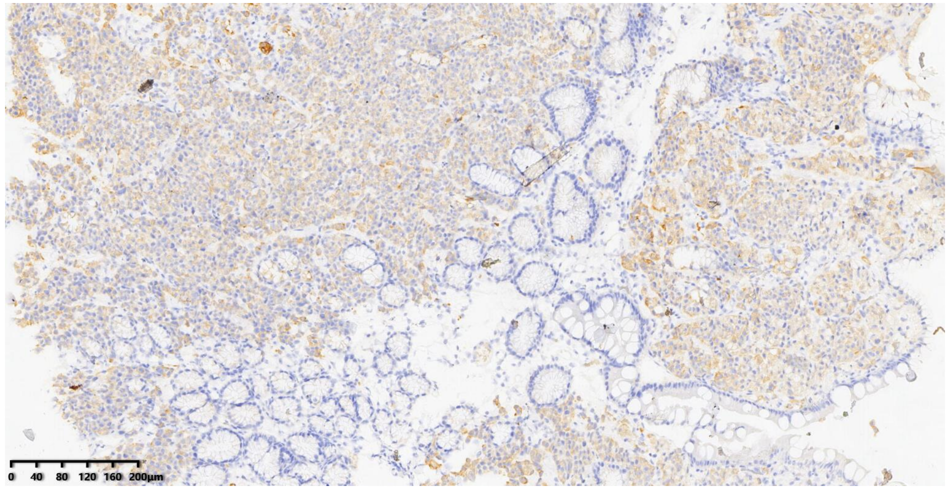

CK7 positive

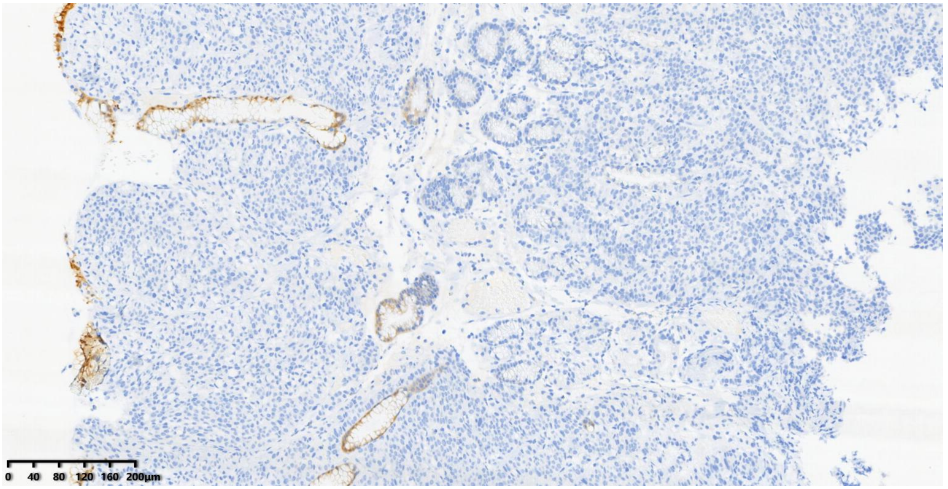

HER-2 negative

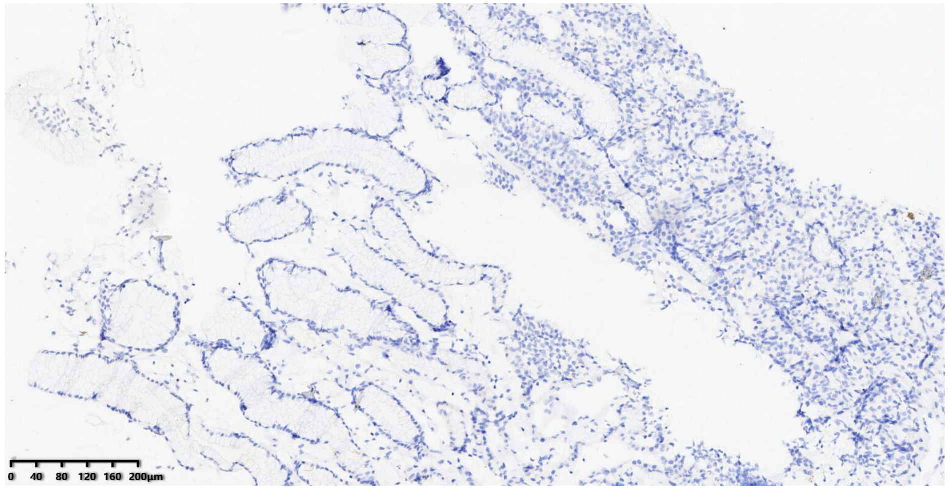

NapsinA negative

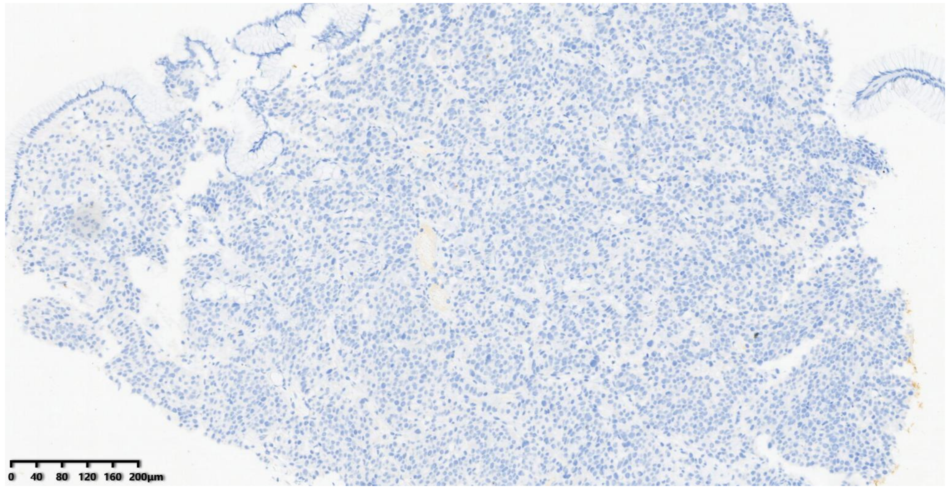

TTF-1 negative

Supplementary Figure 1. Immunohistochemistry results of CK7, HER-2, NapsinA, and TTF-1.
